# p53-mediated adaptation to serine starvation is retained by a common tumour-derived mutant

**DOI:** 10.1186/s40170-018-0191-6

**Published:** 2018-12-03

**Authors:** Timothy J. Humpton, Andreas K. Hock, Oliver D. K. Maddocks, Karen H. Vousden

**Affiliations:** 1 CRUK Beatson Institute, Switchback Road, Glasgow, G61 1BD UK; 2 Francis Crick Institute, 1 Midland Road, London, NW1 1AT UK; 3 Wolfson Wohl Cancer Research Centre, Institute of Cancer Sciences, University of Glasgow, Switchback Road, Glasgow, G61 1QH UK

**Keywords:** p53, Serine starvation, MDM2, Antioxidant

## Abstract

**Background:**

In response to oncogenic stress, the tumour suppressor protein p53 can induce the elimination of cells through induction of cell death or senescence, helping to restrain malignant progression. Conversely, under nutrient stress, p53 can protect cells by supporting metabolic adaptation. Many cancers express mutant p53 proteins that have lost the cell-elimination properties of wild-type p53. However, a previous report showed that a tumour-derived mutant can retain the ability to support cells under glutamine starvation.

**Results:**

We show that a commonly occurring p53 mutant, R248W, retains wild-type ability to support survival under serine starvation. R248W, but not R175H, can engage p21 and MDM2, which both function to limit oxidative stress and facilitate the switch to de novo serine synthesis. In vivo, the growth of R248W-expressing tumours is resistant to dietary depletion of serine and glycine, correlating with an increased capacity to limit ROS compared to tumours expressing R175H. Human cancers expressing this p53 mutant show a worse outcome.

**Conclusion:**

Our work shows that mutant p53s can selectively retain wild-type p53 functions that allow adaptation to serine starvation through the activation of antioxidant defence pathways. Tumours containing this p53 mutation are resistant to serine-limited conditions and less responsive to therapy.

**Electronic supplementary material:**

The online version of this article (10.1186/s40170-018-0191-6) contains supplementary material, which is available to authorized users.

## Background

The p53 protein plays an important role as a tumour suppressor, with loss of wild-type p53 activity evident in many human tumours [1]. The importance of p53’s tumour suppressor role is demonstrated by the consequences of germline loss or mutation of p53, which lead to early tumour development in both humans and mice [2, 3]. Functioning as a transcription factor, p53 regulates a complex network of gene expression that leads to the activation of a variety of responses, including both cell survival and cell elimination [4]. These different and opposing functions of p53 have been rationalised in a model where p53 responds to transient metabolic stress by supporting cell survival and repair, while more severe damage or persistent stress promotes p53 responses that eliminate the affected cell [5]. Of note, however, the survival activities of p53 can support cells under various forms of metabolic stress, and this activity has been linked to increased tumourigenicity and resistance to therapy [6, 7].

Many tumour-associated p53 mutations lead to the expression of full-length p53 proteins carrying single amino acid substitutions, which are generally clustered in the DNA-binding domain of the p53 protein. While almost every amino acid in this region has been found mutated in cancer, a few “hotspot” codons are more frequently mutated, with substitutions of three residues in particular (R175, R248, and R273) accounting for over 15% of p53 mutations across all tumour types [8]. Interestingly, substitutions of amino acids at these residues have different effects on the overall structure of p53. R248 and R273 are both residues of p53 that directly contact DNA and mutations at these sites (most commonly R248Q, R248W, R273H, and R273C) lower the affinity of p53 to DNA and thus impede its transcriptional activity [9]. Residue R175 is not at a DNA contact site, but substitutions at this residue (most commonly R175H) lead to an alteration in the conformation of the p53 protein, again inhibiting DNA binding to consensus p53 response elements [9]. Overall, therefore, these mutations lead to a loss of wild-type p53 function. However, comparison of p53-null and mutant p53 expressing tumours has shown that the expression of mutant p53 can result in a gain of function, leading to a more aggressive phenotype [10, 11] generally associated with an enhanced ability to promote invasion and metastasis [12]. Notably, these “gain of function” properties are not generally exhibited by wild-type p53.

In light of the observation that wild-type p53 can function to support cells under nutrient starvation and that this activity of p53 can support tumour development, we considered the possibility that some mutant p53s may retain the pro-survival activities of wild-type p53 while concomitantly losing the cell elimination functions. We showed previously that wild-type p53 can support the adaptation of cells to serine and glycine starvation by activating the expression of p21, so diverting limiting serine to the synthesis of glutathione for antioxidant defence, rather than to nucleotide synthesis [13]. Another transcriptional target of p53, MDM2, was also shown to help cells under serine starvation by cooperating with ATF4 in the activation of the de novo serine synthesis pathway genes [14]. It is therefore of interest to note that some tumour-derived p53 mutants retain some wild-type transcriptional activity, and interestingly, a recent study has reported cancer cell survival activities of mutant p53 under glutamine starvation that reflected an ability to induce the expression of p21 [15].

## Methods

### Cell culture

Parental HCT116 cells were purchased from ATCC. HCT116 p53 KO clones and p53 KO clones stably expressing p53 mutants R175H, R248W, and R273H were created using CRISPR/Cas9 and subsequent infection with pWZL-p53mut-BLAST and pBabe-iRFP-PURO plasmids as previously described [16]. Stock flasks of all cells were maintained in McCoy’s 5A (modified) medium (Gibco, 26600023) supplemented with 10% FBS and 1% penicillin-streptomycin. All cells were cultured at 37 °C in a humidified atmosphere of 5% CO_2_.

### Serine and glycine deprivation

Serine and glycine deprivation experiments were conducted as previously described [13]. Briefly, cells were seeded and left for 24 h in Dulbecco’s modified Eagle medium (DMEM) (Gibco, 21969) supplemented with 10% FBS and 2 mM L-glutamine. The medium was then changed daily either continuing with the seed DMEM (full medium conditions) or switching to starvation medium (− SG). The − SG medium consisted of MEM (21090) supplemented with additional 1× MEM vitamins (Gibco, 11120), 10% dialysed-FBS (Hyclone, Thermo Scientific), 2 mM L-glutamine, and additional D-glucose (to 25 mM).

### Growth curves

For counting, cells were seeded in 24-well plates in full medium. The next day, cells were shifted to serine- and glycine-depleted medium or given fresh full medium. Medium was replaced every day. For counting, cells were trypsinized, re-suspended in PBS-EDTA, and counted with a CASY Model TT Cell Counter (Innovatis, Roche Applied Science) at the indicated time points in each experiment. Cell counts were conducted prior to any media change.

Measuring cell growth using iRFP was performed as previously described [17]. Briefly, cells were seeded in 96-well CellBIND black microplates with clear flat bottom (Corning 3340) and allowed to settle. The next day, cells were shifted to serine- and glycine-depleted medium or given fresh full medium. Media were replaced daily. iRFP intensity was measured using an Odyssey Li-Cor. For quantification, plates were scanned at 169 μm resolution with a 3.5-mm offset and a low-intensity setting held constant throughout each experiment. Image Studio software (LI-COR, V5.2) was used to scan and subsequently quantify the plates.

### Flow cytometry

Adherent cells were labelled with CellROX Green reagent (5 μM) for 15 min in serum-free and phenol-red-free DMEM (C10444, Life Technologies). Cells were then washed in phosphate buffered saline (PBS) and re-suspended in 1x PBS+ 2%FBS for analysis. 4′,6-Diamidino-2-phenylindole dihydrochloride (DAPI, Sigma Aldrich) was added to a final concentration of 1 μg/ml to each sample and was used to identify viable cells for analysis. Single cells were analysed on a BD Fortessa flow cytometer using unstained and cumene hydroperoxide-stained (100 μM) cells as controls. At least 10,000 events were collected for each sample. Data were analysed using FlowJo X 10.0.7r2 (FlowJo, LLC). Unless otherwise stated, median fluorescence intensity values were obtained and compared across samples.

### siRNA transfection

The siRNA used to target human *MDM2*, *p21 (CDKN1A)*, and *ATF4*, as well as the non-targeting siRNA control pool, were all purchased from Dharmacon (siGENOME SMART pool siRNA) and transfected at 20 nM concentration using Lullaby siRNA transfection reagent and the manufacturer’s recommended reverse transfection procedure (OZ Biosciences). Cells were left in the seeding/transfection medium for 24 h prior to any medium change.

### Liquid chromatography-mass spectrometry

Liquid chromatography-mass spectrometry (LC-MS) sample preparation and analysis were performed broadly as described previously [18, 19]. Cells were incubated in fresh assay medium for 3 h. Metabolites were extracted by rapidly removing cell media, washing wells once with ice-cold PBS, and lysing cells in ice-cold methanol/acetonitrile/H2O (50:30:20) at volumes scaled based on cell counts of the counting plate to 2 × 10^6^ cells per millilitre extraction buffer. Sample plates were shaken at 4 °C for 10 min before the extraction buffer was collected from each well, spun for 15 min at 16,000×*g* in a chilled (4 °C) centrifuge, and then analysed by LC-MS.

For metabolite analysis, a Q Exactive Orbitrap mass spectrometer (Thermo Scientific, Waltham, MA, USA) was used together with a Thermo Ultimate 3000 HPLC system. The HPLC setup consisted of a ZIC-pHILIC column (SeQuant, 150 × 2.1 mm, 5 μm, Merck KGaA, Darmstadt, Germany), with a ZIC-pHILIC guard column (SeQuant, 20 × 2.1 mm) and an initial mobile phase of 20% 20 mM ammonium carbonate, pH 9.4, and 80% acetonitrile. Cell and media extracts (5 μl) were injected, and metabolites were separated over a 15-min mobile phase gradient, decreasing the acetonitrile content to 20%, at a flow rate of 200 μl/min and a column temperature of 45 °C. The total analysis time was 23 mins. All metabolites were detected across a mass range of 75–1000 m/z using the Q Exactive mass spectrometer at a resolution of 35,000 (at 200 m/z), with electrospray (ESI) ionisation and polarity switching to enable both positive and negative ions to be determined in the same run. Lock masses were used, and the mass accuracy obtained for all metabolites was below 5 ppm. Data were acquired with Thermo Xcalibur software. The peak areas of different metabolites were determined using Thermo TraceFinder 4.0 software where metabolites were identified by the exact mass of the singly charged ion and by known retention time on the HPLC column. Commercial standards of all metabolites detected had been analysed previously on this LC-MS system with the pHILIC column.

### Immunoprecipitation

For the analysis of p53 conformation, IP experiments were performed broadly as previously described [20]. Adherent cells were washed once in ice-cold PBS. Protein lysates were then prepared using RIPA buffer (Millipore) supplemented with cOmplete ULTRA EDTA-free protease inhibitors (Roche) and PhosSTOP phosphatase inhibitors (Roche). Equivalent amounts of total protein (1.5–2 μg), determined using a Pierce BCA protein assay kit (ThermoFisher Scientific), were incubated overnight at 4 °C with either p53 Ab1620 (Abcam) or pAb240 (Santa Cruz Biotechnology) antibody (1:100 dilution) and 20 μl of Protein G Dynabeads (ThermoFisher Scientific). Beads were washed three times in RIPA and resuspended in buffer containing RIPA, NuPAGE LDS sample buffer, and NuPage Reducing Agent (both ThermoFisher Scientific). Protein was eluted from the beads by boiling at 95 °C for 10 min. The resulting samples were analysed by Western blotting.

For ATF4 IP experiments, samples were prepared as described above except they were incubated with ATF4 antibody D4B8 (Cell Signaling Technology) (1:100 dilution) instead of the p53 antibodies.

### Western blotting

As with the IP experiments, protein lysates were prepared using RIPA buffer (Millipore) supplemented with cOmplete ULTRA EDTA-free protease inhibitors (Roche) and PhosSTOP phosphatase inhibitors (Roche). The resulting samples were separated using precast NuPAGE 4–12% Bis-Tris protein gels (ThermoFisher Scientific), transferred to nitrocellulose membranes using NuPAGE transfer buffer (ThermoFisher Scientific) with 20% methanol, and blocked in a PBS solution containing 5% BSA (Sigma Aldrich) and Tween-20 (Sigma Aldrich). Membranes were incubated overnight at 4 °C with primary antibodies (1:1000 dilution unless otherwise indicated). Membranes were washed in PBS-Tween20 and incubated with secondary antibodies (1:15000 dilution) for 45 min at room temperature prior to a final set of washes in PBS (no Tween 20) and detection. Proteins were detected using a Li-Cor Odyssey Infrared Scanner and LiCor Image Studio Software.

#### Primary antibodies

MDM2 (SMP-14) (sc-965), MDM2 (D-7)(sc-13161), CREB-2 (B-3)(sc-390063), p53 (DO-1) (sc-126), PSPH (H-11)(sc-365183), PSAT1 (L-24)(sc-133929), p53 (pAb240) (sc-99), and Actin (I-19)(sc-1616) were purchased from Santa Cruz Biotechnology.

The p53 (pAb1620) (ab16776) was purchased from Abcam.

COXIV (#11967), HSP90 (#4877), p21 (#2947), PHGDH (#66350), ATF4 (#11815), and HA-tag (#3724) were purchased from Cell Signaling Technology.

#### Secondary antibodies

IRDye 800CW and 680LT all raised in Donkey (anti-Goat, anti-Rabbit, and anti-Mouse) were used for standard Western blots (LiCor).

Light chain-specific monoclonal antibodies with AF680 or AF790 probes were used for IP Western blots (Jackson ImmunoResearch).

### Animal experiments

Mouse procedures were carried out under UK Home Office licence number 60/4181 (Karen Blyth) and conducted in line with the Animals (Scientific Procedures) Act 1986 and the EU Directive 2010. Experiments were sanctioned by Local Ethical Review Process (University of Glasgow). Mice were housed on a 12/12 light/dark cycle and fed and watered ad libitum.

For HCT116 xenograft experiments, athymic female nude (nu/nu) mice (obtained from The Jackson Laboratory, 7–8 weeks old) were fed specified diets either containing or lacking serine and glycine as previously described [13, 21] for a period of 1 week prior to the start of the experiment. Mice in each group received bilateral subcutaneous injections of 100 μl of either HCT116 R175H or R248W cells (2 × 10^6^ cells) suspended in phosphate buffered saline (PBS) (*n* = 5 mice per group). Each mouse was injected with only one cell line. Following injection, subcutaneous tumour growth was monitored by both iRFP imaging as previously described [22] and using callipers (both twice per week). Relative tumour growth was determined by comparing iRFP intensity to the baseline (day 3 post-injection) scan for each tumour. Tumour volume by callipers was calculated using the formula (length × width^2^/2).

### Immunohistochemistry

Tissues were fixed in 10% neutral buffered formalin for 24 h before paraffin embedding and sectioning. Slides cut from paraffin blocks were de-paraffinised and rehydrated. Antigen retrieval was performed in citrate-based antigen unmasking solution (Vector H-3300) for 15 min in a microwave. Endogenous peroxidase activity was quenched by incubation with BLOXALL blocking solution (Vector SP-6000) according to the manufacturer’s instructions. For p21 and MDA staining, slides were blocked in 5% BSA with 5% rabbit serum in TBS-T for 1 h. Slides were incubated with primary antibody diluted to 1:1000 in blocking solution overnight at 4 °C. Secondary antibody incubation and downstream signal detection were performed using the Vectastain ABC elite kit (Vector PK-6010) and ImmPACT DAB (Vector SK-4105) following the manufacturer’s recommendations. For MDM2 IHC staining, slides were instead processed using the M.O.M. kit and protocol from Vector Labs (BMK-2202) with the primary antibody diluted to 1:1000. Slides were dehydrated, counterstained, and mounted with coverslips prior to analysis using Olympus BX51 microscope.

### IHC quantification

Seven random × 20 magnification images were taken from each IHC slide using an Olympus BX51 microscope with Zen Blue software (Zeiss). From these images, the positive staining per slide area was calculated using ImageJ software. Each image was expanded into an RGB stack with the green channel used for quantification as it offered the best separation for DAB staining. For each set of stained slides, a control slide was used to set a threshold for positive staining. This threshold was then applied in batch format to the rest of the images in the set (all images from all slides stained with a given antibody), and the resulting positive staining area for each was measured and collected by ImageJ. These measured areas were then averaged across the images of a given slide to give a staining percentage area value for each sample.

### Human patient survival analysis

We obtained survival data by accessing the publicly available harmonised cancer datasets hosted on the National Cancer Institute GDC data portal. Through the portal, we compared survival between patients with R248 mutations (either R248W or R248Q) to those with the R175H mutation. Patient survival data arising from this search were imported into GraphPad Prism. The median survival of each group was compared using the Mantel-Cox Log-rank test.

### Data plotting and statistical analysis

All data were plotted using Prism 7 (Graph Pad). Statistical analysis for each experiment was performed using the tools within Prism 7 and the indicated tests.

## Results

**Fig. 1:**
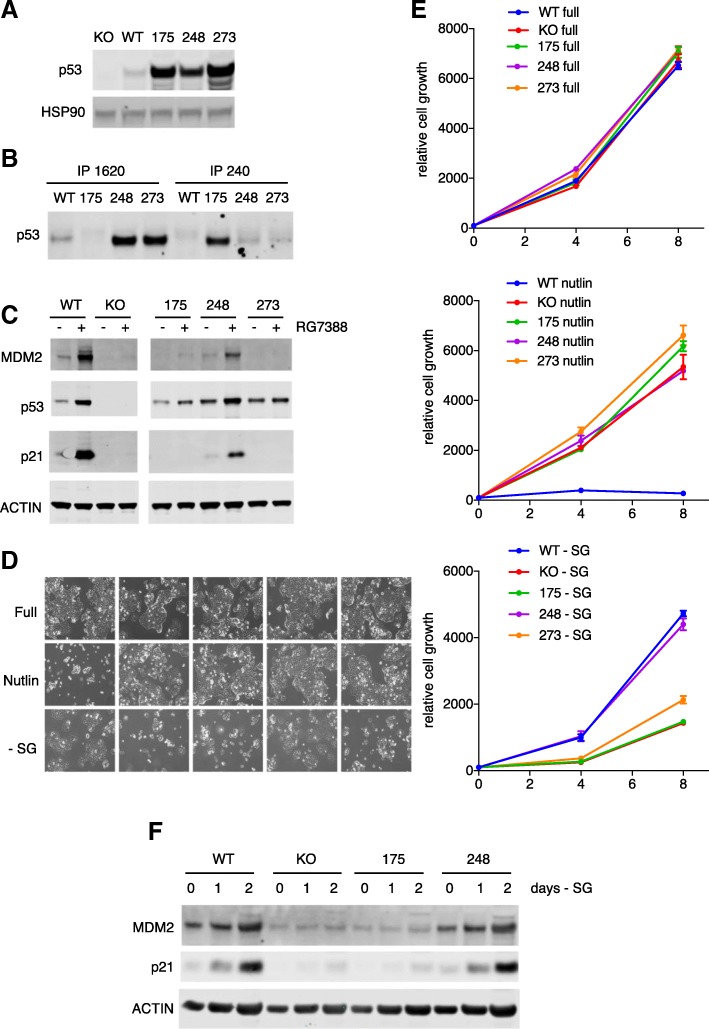
The R248W mutant retains wild-type ability to engage p21 and MDM2 and promotes adaptation to serine and glycine (SG) deprivation. **a** Western blot for p53 in HCT116 cells with parental WT p53 (WT), CRISPR-mediated p53 deletion (KO), or p53 KO cells reconstituted with mutant p53 expression constructs R175H, R248W, or R273H (175, 248, 273). HSP90 expression used as a loading control. **b** p53 protein in wild-type (IP by pAB1620) and mutant/unfolded (IP by pAb240) conformation in the indicated cell lines, detected by immunoprecipitation followed by Western blot. **c** Western blot for p53, MDM2, and p21 in the indicated cell lines treated with the MDM2 inhibitor RG7388 (+) (5 μM) or DMSO vehicle control (−) for 24 h. **d** The indicated cell lines were cultured in full medium, full medium containing the MDM2 inhibitor nutlin-3A (10 μM), or SG-depleted medium for 8 days. **e** Cell counts of the indicated cell lines cultured in full medium, full medium containing the MDM2 inhibitor nutlin-3A (10 μM), or SG-depleted medium for 8 days. Data are represented as the mean of triplicate wells ± SEM for each condition. **f** Western blot for MDM2 and p21 in the indicated cell lines cultured in full medium (day 0) before being switched to grow in SG-depleted medium for 1 or 2 days. Note: this blot was re-probed for PSAT-1 and PSPH and shown in Fig. 2c. The ACTIN loading control blot is therefore the same in both figures

Based on our previous observations showing a role for wild-type p53 in the adaptation to serine and glycine deprivation [13], we expanded our analysis to determine whether common p53 mutant alleles also retain the ability to support cells under serine and glycine starvation. A previously described series of HCT116 cells (a human colorectal tumour line) that express wild-type p53, no p53, or the R248W p53 mutant [23] are complicated by potential activity arising from high levels of expression of a smaller p53 isoform in the line deleted for full-length p53 [24, 25]. To avoid this issue and to develop a model in which additional p53 mutants could be examined, we used gene editing to produce a p53 null line that lacked any detectable p53 expression and then reintroduced ectopic expression of three common p53 mutants —R175H, R248W, and R273H. As seen in human cancers [26], the mutant forms of p53 were expressed at higher levels than the endogenous wild-type p53 (Fig. 1a). The R175H mutation has been reported to drive a conformational shift in the p53 protein, while R248W and R273H alter DNA-contacting residues and retain a predominantly wild-type conformation [27, 28]. Using conformation-specific antibodies, we were able to confirm that in our engineered cells, the R175H mutant adopts an unfolded or mutant conformation (detected by Ab240) while the R248W and R273H mutants remained mostly in a wild-type conformation (detected by Ab1620) (Fig. 1b). The retention of the wild-type conformation by R248W and R273H prompted us to test whether these mutant p53s retained any ability to induce the expression of canonical p53 target genes. Although the basal levels of the mutant p53 proteins were higher than that of the wild-type p53, all p53 proteins were somewhat stabilised in response to treatment with the MDM2 inhibitor RG7388 [29], reflecting an ability of MDM2 to target both wild-type and mutant p53s for degradation [30, 31]. This stabilisation was accompanied by increased expression of two canonical p53 target genes, p21 and MDM2, in both wild-type p53 and R248W-expressing cells, although this response was not seen in R175H- or R273H- expressing cells (Fig. 1c).

Proliferation assays in normal medium showed a similar growth rate for each of these cell lines, regardless of p53 status (Fig. 1d, e). Activation of p53 using the MDM2 inhibitor nutlin-3A blocked proliferation in the wild-type p53-expressing cells, but not the p53 null or R248W cells (Fig. 1d, e), despite the induction of clearly detectable levels of p21 in the R248W cells. However, the p53 null cells were more sensitive than wild-type p53 cells to removal of serine and glycine from the growth medium, confirming previous observations [13].

Interestingly, cells expressing R248W retained the ability to proliferate in serine- and glycine-free medium while R175H-expressing cells were less able to adapt to these conditions, behaving comparably to p53 null cells (Fig. 1d, e). Further analysis of the previously published HCT116 cell lines confirmed the ability of R248W to function like wild-type p53 in supporting proliferation in the absence of serine and glycine (Additional file 1: Figure S1A). Importantly, serine and glycine starvation induced p53 activity, as measured by the induction of p21 and MDM2 expression, in both the wild-type p53 and R248W cells, but not p53 null or R175H cells (Fig. 1f).

**Fig. 2:**
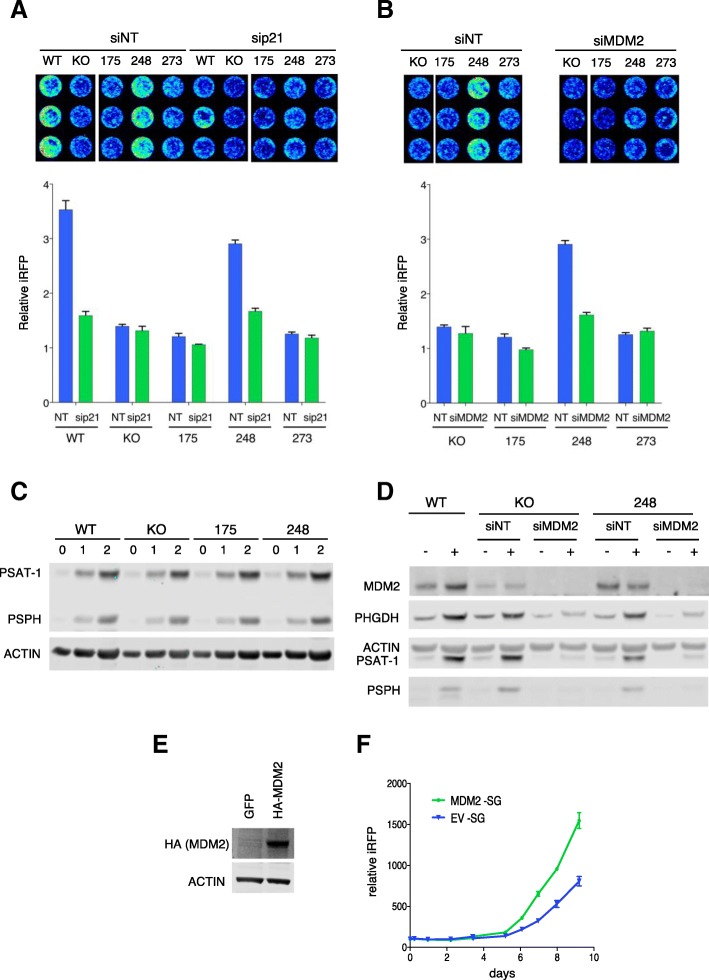
Augmented expression of p21 and MDM2 during serine and glycine (SG)-deprivation promotes adaptation in R248W cells independently of MDM2-facilitated SSP induction. **a** Growth of the indicated cell lines treated with non-targeting control or p21 siRNA and cultured in SG-depleted medium for 7 days. iRFP intensity in each well on day 7 (top) and iRFP level per well relative to the day 2 reading (bottom) shown for each cell line. Data are represented as the mean of triplicate wells ± SEM for each condition. **b** Growth of the indicated cell lines treated with non-targeting control or MDM2 siRNA and cultured in SG-depleted medium for 7 days as in (**a**). **c** Western blot PSAT-1 and PSPH in the indicated cell lines cultured in SG-depleted medium for 1 or 2 days. Note: this is a re-probe of the blot shown in Fig. 1f, where MDM2 and p21 levels are shown. The ACTIN loading control blot is therefore the same in both figures. **d** Western blot for SSP enzymes PSAT-1, PSPH and PHGDH, and MDM2 in the indicated cell lines treated with non-targeting control or MDM2 siRNA and grown in SG-depleted medium for 2 days. **e** Western blot for HA-MDM2 in p53 KO cells transfected with an HA-tagged MDM2 expression construct (HA-MDM2) or a control vector containing GFP. **f** Growth of p53 KO cells expressing HA-tagged MDM2 or a control vector (EV) and cultured in SG-depleted medium for 9 days. Data are presented as mean ± SEM and depicted as iRFP intensity relative to the initial measurement obtained from triplicate wells per condition.

Previous studies have shown that both p21 and MDM2 can contribute to the ability of cells to adapt to serine starvation [13, 14]. Consistently, siRNA-mediated depletion of p21 inhibited the proliferation of wild-type p53 and R248W-expressing cells under serine and glycine starvation without impacting the growth of these cells in fully fed conditions (Fig. 2a and Additional file 1: Figure S2A). In full medium, depletion of MDM2 in wild-type p53 cells inhibited growth through stabilization of p53, while MDM2 inhibition did not impact the growth of R248W expressing cells (Fig. 1c) under these conditions. However, depletion of MDM2 strongly decreased the growth of R248W cells (Fig. 2b and Additional file 1: Figure S2B) in serine- and glycine-free medium, consistent with a role for MDM2 in allowing the adaptation of these cells to loss of exogenous serine and glycine.

Previous studies have shown that during serine starvation, MDM2 drives the expression of the serine synthesis pathway enzymes through interaction with ATF4 [14], so facilitating proliferation. We therefore considered whether the lack of induction of MDM2 in p53 null or R175H-expressing cells may hinder the upregulation of the serine synthesis pathway (SSP). As expected, expression of PSAT-1 and PSPH were efficiently activated in response to serine starvation in p53 wild type and R248W cells (Fig. 2c). However, equally efficient induction of these SSP genes was also seen in p53 null and R175H cells (Fig. 2c). As already shown, p53 null and R175H cells expressed lower levels of MDM2 that was not induced by serine starvation. However, directly depleting cells of MDM2 using siRNA substantially inhibited the increased expression of PHGDH, PSAT-1, and PSPH in both p53 null and R248W cells (Fig. 2d) under serine starvation. These results support the importance of MDM2 in allowing activation of the SSP but suggest that even the low levels of MDM2 retained in p53 null cells are sufficient for this activity.

In order to assess whether the p53-dependent differences in MDM2 levels seen in the cell lines contribute to the ability of these cells to proliferate in the absence of exogenous serine, we introduced a plasmid to express MDM2 into p53 null cells. In this system, ectopic MDM2 expression did not affect the growth rate of cells in fully fed conditions, but clearly supported enhanced proliferation under serine and glycine starvation (Fig. 2e, f and Additional file 1: Figure S2C). In contrast, ectopic expression of MDM2 in cells containing wild-type p53 did not further increase their ability to adapt to serine and glycine starvation (Additional file 1: Figure S2D). Taken together, the data suggest that the p53-dependent increase in MDM2 above basal levels seen in wild-type p53 and R248W cells contributes to their ability to adapt to serine and glycine starvation through a mechanism that is distinct from the requirement for MDM2 to induce the SSP.

**Fig. 3:**
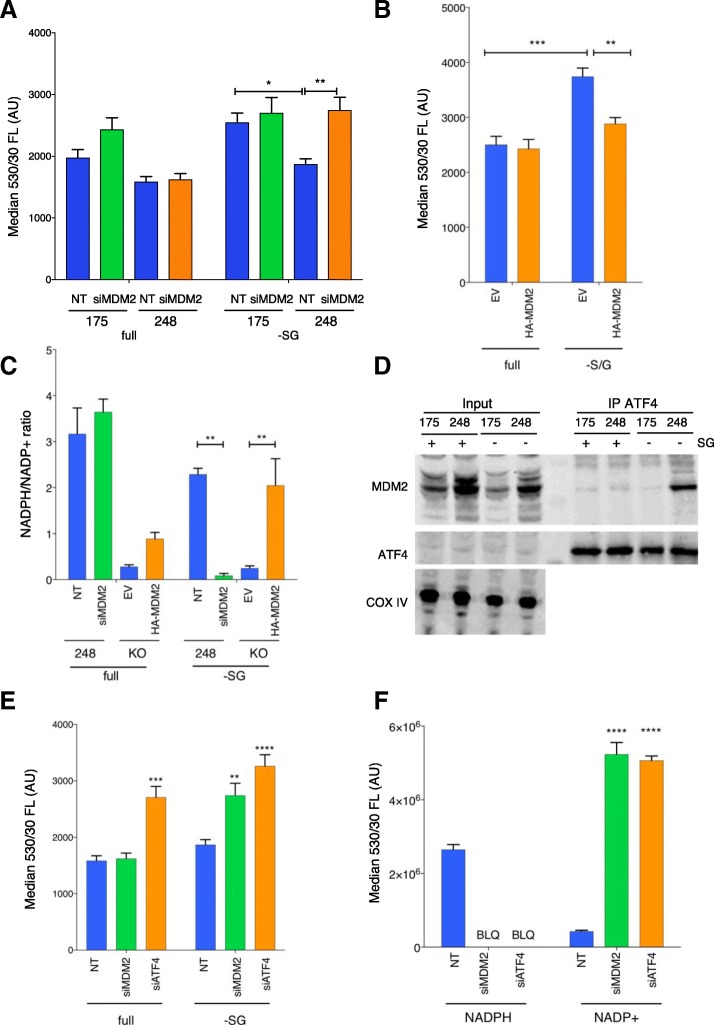
MDM2 supports redox robustness and interacts with ATF4 in R248W cells undergoing serine and glycine (SG) deprivation. **a** Oxidative stress levels in the indicated cell lines treated with non-targeting control or MDM2 siRNA and cultured in SG-depleted or full medium for 3 days. Median fluorescent intensity of the CellRox Green probe in triplicate samples is presented as mean ± SEM per condition. Data analysed using a two-way ANOVA with Holm-Sidak’s multiple comparisons test and multiplicity-adjusted *p* values. **b** Oxidative stress levels in the indicated cell lines expressing HA-tagged MDM2 or a control vector (EV). **c** NADPH/NADP+ ratio in R248W cells with or without siRNA depletion of MDM2, or in p53 null cells expressing HA-MDM2 or empty vector (EV), and cultured in SG-depleted or full medium for 3 days. The NADPH/NADP+ ratio is shown as mean ± SEM from triplicate wells per condition. Data were analysed using a two-way ANOVA with Holm-Sidak’s multiple comparisons test and multiplicity-adjusted *p* values. **d** IP and Western blot of the indicated cell lines cultured in SG-depleted or full medium for 24 h. ATF4 was immunoprecipitated from each sample, then the imunoprecipitate (IP) or input lysate probed by Western blot for MDM2 and ATF4. **e** Oxidative stress levels in R248W cells treated with non-targeting control, MDM2, or ATF4 siRNA as in (**a**). **f** Total NADPH and NADP+ pools in R248W cells treated with non-targeting control (NT), MDM2, or ATF4 siRNA as in (**c**). BLQ: metabolite peak below quantitation/detection limit in the experiment. Stars indicate statistical significance.

We next considered whether other consequences of the MDM2/ATF4 interaction may be contributing to the ability of increased MDM2 to protect cells under serine and glycine starvation. Previous work demonstrated the importance of ROS limitation when cells are serine starved [13], and ATF4 has been shown to play a role in supporting an antioxidant response [32–34]. R248W cells showed a trend towards lower ROS levels than R175H cells in full medium and were able to limit ROS accumulation more efficiently than R175H cells under serine and glycine starvation (Fig. 3a). However, this ability of R248W cells to limit ROS accumulation upon serine/glycine starvation was lost following depletion of MDM2 (Fig. 3a). Conversely, overexpression of MDM2 in p53 null cells substantially prevented the elevation in ROS levels seen following serine and glycine starvation (Fig. 3b). Consistent with this observation, R248W cells maintained a more reduced NADPH/NADP+ ratio than p53 null cells under both fully fed and serine and glycine starved conditions (Fig. 3c). This advantage was eliminated in R248W cells following depletion of MDM2 (Fig. 3c), while ectopic expression of MDM2 in p53 null cells resulted in a recovery of the NADPH/NADP+ ratio (Fig. 3c). Interestingly, we detected a substantial increase in the formation of an MDM2/ATF4 complex in R248W cells undergoing serine and glycine starvation, which was not detected in R175H-expressing cells (Fig. 3d). Building on these findings, we sought to establish the importance of ATF4 for the redox response of R248W cells. We observed increased ROS levels and a greatly diminished NADPH/NADP^+^ ratio during serine and glycine deprivation of R248W cells depleted of ATF4 (Fig. 3e, f). Interestingly, while depletion of MDM2 did not affect ROS levels or NADPH/NADP^+^ ratios in R248W cells under fully fed conditions (Fig. 3a, c, e), ATF4 function was required to limit ROS under both fully fed and serine and glycine starved conditions (Fig. 3e). Taken together, these findings suggest that enhanced redox robustness, facilitated in part by increasing the previously published interaction between ATF4 and MDM2 [14], helps to support proliferation in R248W cells under serine and glycine starvation.

**Fig. 4:**
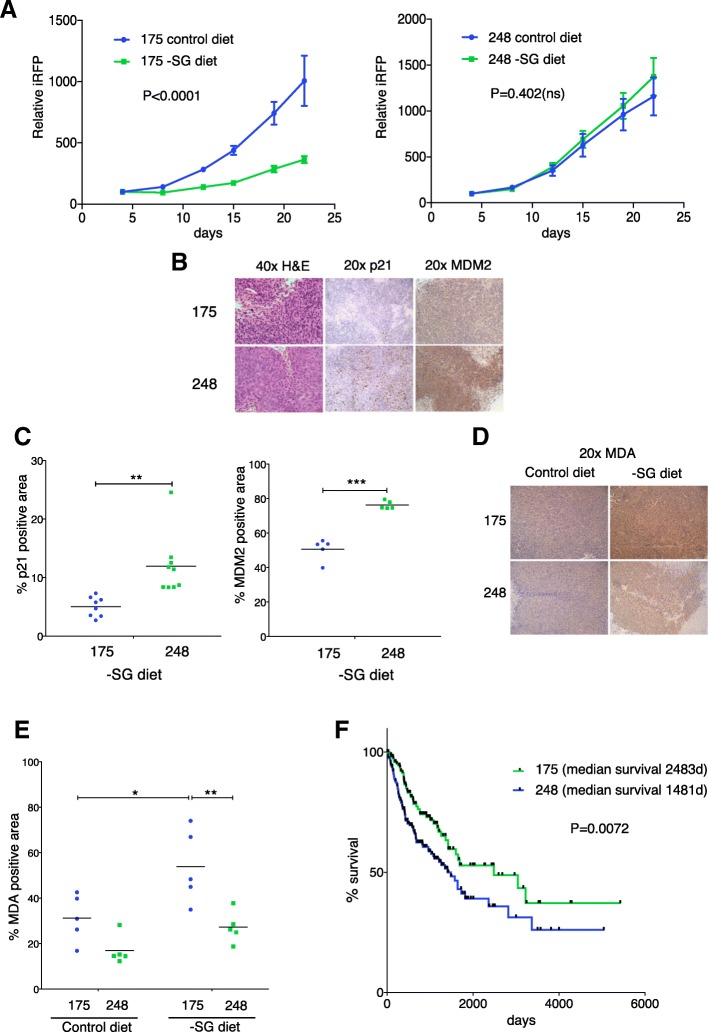
R248W mutant p53 confers enhanced tumour growth and increased ROS control in serine- and glycine (SG)-depleted conditions in vivo, correlating with decreased human patient survival. **a** R175H or R248W tumour growth in SG or control fed mice. Data presented as iRFP level relative to day 4 baseline reading (mean ± SEM) from *N* = 9 R175H control, *N* = 8 R175H − SG, *N* = 8 R248W control, and *N* = 10 R248W − SG tumours. Data analysed using a two-way ANOVA with Tukey’s multiple comparisons test and multiplicity-adjusted *p* values. *p* value reported in figure from final measurements. **b** H&E staining (× 40 magnification) and p21 and MDM2 IHC staining (× 20 magnification) in tumours from -SG fed mice. Representative images from at least *N* = 5 tumours per group. **c** Quantification of p21 and MDM2 IHC staining in tumours from -SG fed mice. Each point represents the mean positive area taken from seven random images per tumour. The black bar represents the mean value per group. *N* = 9 tumours per group for p21 IHC and *N* = 5 tumours per group for MDM2 IHC. Data analysed using a paired *t* test for each stain. **d** Malondialdehyde (MDA) IHC staining (× 20 magnification) in tumours from control or − SG fed mice. Representative images shown from *N* = 5 tumours per group. **e** Quantification of MDA IHC staining in tumours from control or − SG fed mice. Each point represents the mean positive area taken from seven random images per tumour. The black bar represents the mean value per group of *N* = 5 tumours. Data were analysed using a two-way ANOVA with Holm-Sidak’s multiple comparisons test and multiplicity-adjusted *p* values. **f** Survival curves for patients whose cancers contained R175H (*N* = 156 cases) or R248 (both W and Q pooled) (*N* = 207 cases). Median survival (2483 days for R175H and 1481 days for R248) was compared between the groups using the Mantel-Cox Log-rank test

Our results show that in cultured cells, the R248W mutant functions like wild-type p53 in supporting cells during serine and glycine starvation. To understand the implication of these observations in vivo, we carried out xenograft studies using R175H and R248W cells, feeding mice with a serine- and glycine-deficient diet [21]. As seen previously with p53 null tumours, the growth of the R175H tumours was retarded in mice on a serine- and glycine-free diet (Fig. 4a and Additional file 1: Figure S3). In contrast, R248W tumours grew equally well regardless of the diet (Fig. 4a and Additional file 1: Figure S3). Immunohistochemical analysis recapitulated the observations in cells, confirming an increased expression of both MDM2 and p21 in the R248W tumours compared to R175H (Fig. 4b, c). Importantly, while R175H tumours showed a clear increase in MDA staining, a marker of lipid peroxidation and oxidative stress, this increase was not seen in the R248W tumours in mice fed on a serine- and glycine-free diet, which were able to sustain redox balance (Fig. 4d, e).

Finally, we hypothesised that the in vitro and in vivo redox advantages we uncovered for HCT116 R248W cells would translate into differences in human patient outcome based on p53 mutational status. To assess this possibility, we utilised the publicly available harmonised cancer datasets hosted on the National Cancer Institute GDC data portal to compare survival between patients with R248 mutations to those with the R175H mutation. Based on this analysis, we found that cases with tumours containing R248 mutations had significantly shorter survival than those with tumours containing the R175H mutation (median survival of 1481 days vs. 2483 days, Fig. 4f). These observations support a previous study showing reduced survival of patients carrying R248 mutants compared to patients with nonsense mutations [35]. Combined with our other observations, we speculate that at least some of the accelerated lethality of R248 tumours can be explained by an enhanced redox robustness and ability to respond to metabolic stress conferred by the retention of pro-survival wild-type p53 functions in p53 R248 mutant cells.

## Discussion

While the selection for p53 point mutations in cancer may reflect the loss of tumour suppressor properties and acquisition of gain of oncogenic functions, it is also possible that some of these p53 mutants may contribute to cancer development by the selective retention of wild-type activities that promote cell survival. We show here that one of the most common hotspot p53 mutants—R248W—retains the ability of p53 wild-type cells to adapt to serine and glycine starvation. This mutant has been associated with a gain of function reflecting the ability to induce expression of drug metabolising enzymes [35] and has also been shown to function in promoting survival of cells undergoing glutamine starvation [15]. We show that serine starvation also leads to the induction of p21 in R248W-expressing cells, and while this may contribute to the adaptation to serine starvation, it is not sufficient to induce an anti-proliferative effect in our system.

MDM2 expression is required for cells to induce SSP enzymes following serine starvation, but HCT116 cells did not depend on a p53-driven increase in MDM2 levels to support SSP activation. However, our study reveals a role for p53-induced MDM2 in protecting cells under serine starvation by ROS regulation. The increase in MDM2 levels seen in R248W p53 cells enhanced the formation of a complex with ATF4 and the induction of an antioxidant response that protected cells from the increase in ROS encountered during the switch to endogenous serine synthesis [13]. Our results illustrate how different levels of MDM2 are necessary for different functions—while very low basal MDM2 levels are sufficient for activation of the SSP, enhanced MDM2 expression that is dependent on wild type or R248W p53 expression correlated with an increase in binding to ATF4 and the induction of robust antioxidant activity.

Clearly, the protective and adaptive functions of p53 can help to support tumour development under conditions of nutrient deprivation. The R248W mutant combines loss of cell elimination functions of wild-type p53 with the retention of the adaptive functions, so offering maximum support for tumour development. Consistently, we see that tumours expressing this mutant are not inhibited by a reduction in circulating serine and glycine (in serine and glycine free diet fed mice) and that expression of this mutant in human tumours is associated with a worse prognosis when compared to p53 null tumours. Several other p53 activities such as the transient induction of autophagy and cell cycle arrest also are likely to help cells adapt to limited exogenous nutrient availability and may be retained by some tumour-derived mutants. We therefore propose that the selection for the expression of p53 point mutants could reflect the retention of some wild-type p53 activity, as well as the previously documented gain of function.

## Conclusions

We show that the tumour-derived p53 R248W mutant can support adaptation to serine starvation through the activation of antioxidant defence pathways. This function reflects the selective retention of one arm of the p53 response in concert with loss of the cell elimination functions of the wild type protein. p53 mutants with this activity give rise to more aggressive tumours that are resistant to dietary serine modulation.

## Additional file

Additional file 1: Supplementary supporting data. (PDF 284 kb)

## Abbreviations

ATF4: Activating transcription factor 4
DMEM: Dulbecco’s modified Eagle medium
FBS: Fetal bovine serum
IHC: Immunohistochemistry
MDA: Malondialdehyde
MDM2: Murine double minute 2
PHGDH: Phosphoglycerate dehydrogenase
PSAT-1: Phosphoserine aminotransferase-1
PSPH: Phosphoserine phosphatase
ROS: Reactive oxygen species
SSP: Serine synthesis pathway
