# Supplementary material for: p53-mediated adaptation to serine starvation is retained by a common tumour-derived mutant
Source: Cancer Metab. 2018 Dec 3;6:18. doi: 10.1186/s40170-018-0191-6 (PMC6276204; doi:10.1186/s40170-018-0191-6)
Supplement: Supplementary file 1 — Supplementary supporting data. (PDF 284 kb) [file 40170_2018_191_MOESM1_ESM.pdf]

A

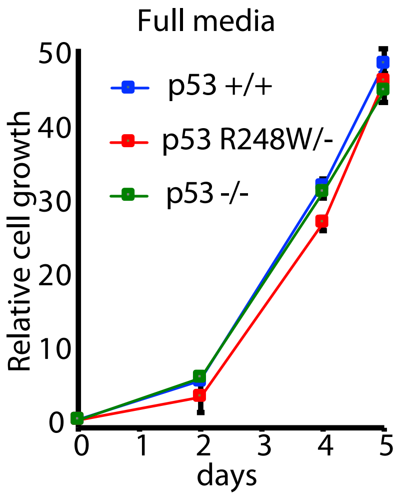

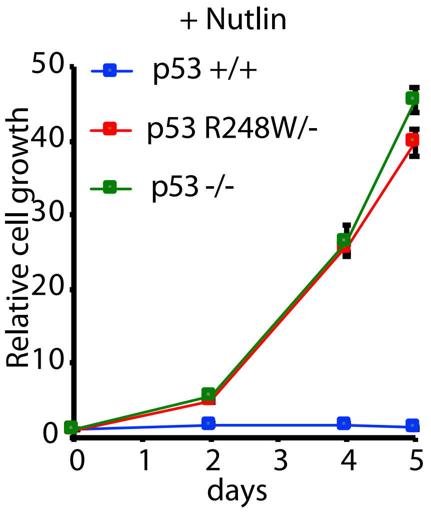

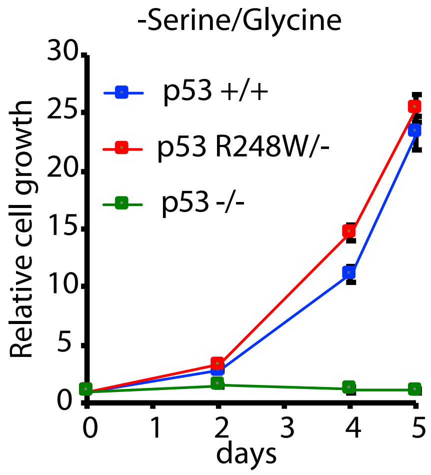

Figure S1: Vogelstein HCT116 cells expressing WT or R248W p53 adapt to serine and glycine deprivation

A. Cell growth in Vogelstein HCT116 cells expressing WT p53, p53-null cells (KO), and the R248W/- p53 mutant cell line cultured in full media, full media containing the MDM2 inhibitor Nutlin-3A (10 µM), or serine and glycine-depleted media for 5 days. Total cell numbers were counted from triplicate wells per condition on days 2, 4, and 5 and tracked as cell growth relative to day zero counts. Data are represented as the mean of triplicate wells ± SEM for each condition. Representative experiment shown.

Sup. Figure 2

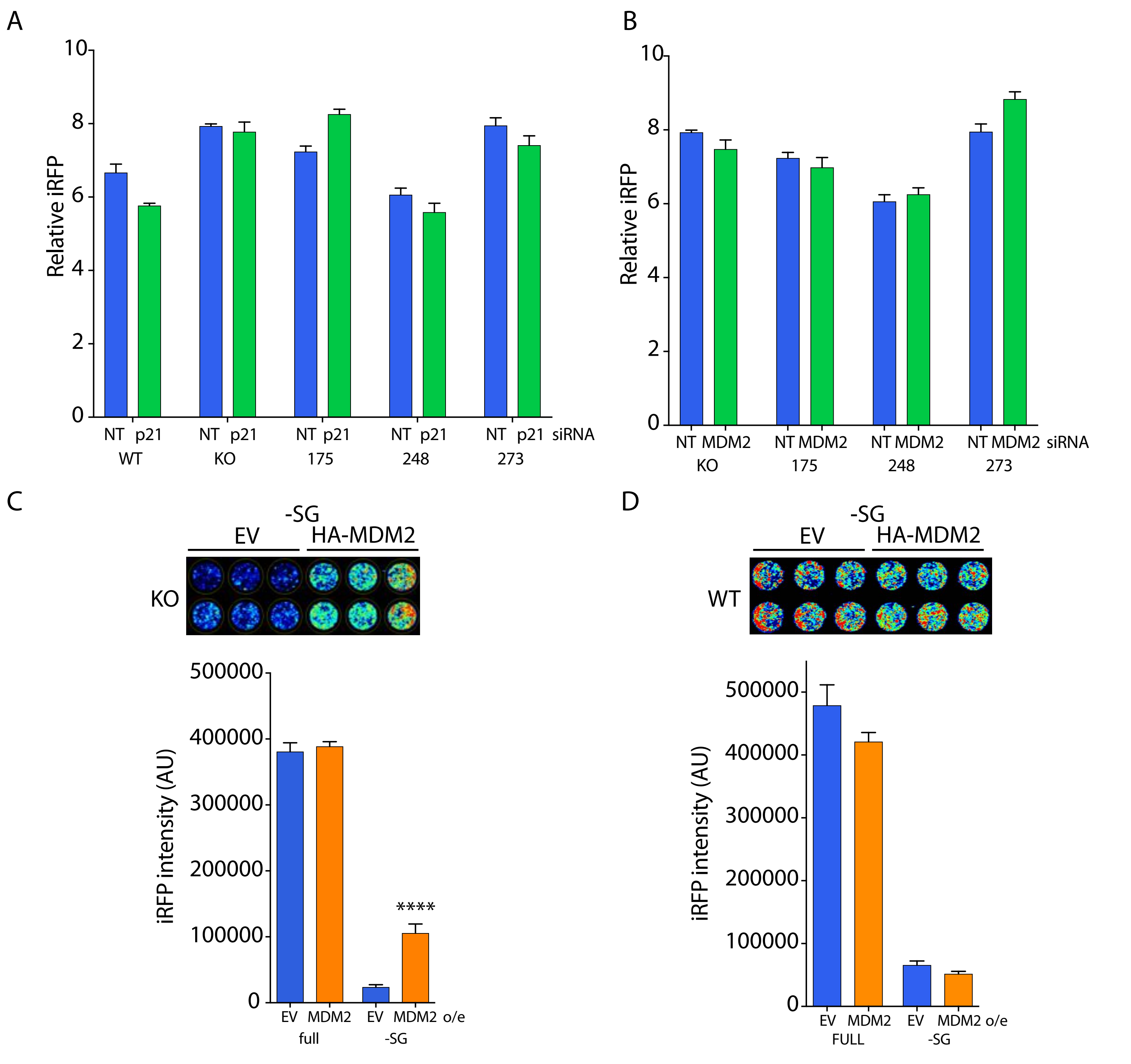

Figure S2: Expression of p21 or MDM2 does not alter cell growth in full media conditions and ectopic MDM2 does not further enhance growth of p53 WT cells undergoing serine and glycine deprivation

A. Cell growth in HCT116 cells expressing WT, KO, R175H, R248W, or R273H p53 treated with non-targeting control (NT) or p21 siRNA and cultured in full media for 5 days. iRFP level per well relative to the day 2 reading shown for each cell line. Data are represented as the mean of triplicate wells ± SEM for each condition. Representative experiment shown.

B. Cell growth in HCT116 cells without p53 (KO), or expressing R175H, R248W, or R273H p53 treated with non-targeting control (NT) or MDM2 siRNA and cultured in full media for 5 days. iRFP level per well relative to the day 2 reading shown for each cell line. Data are represented as the mean of triplicate wells ± SEM for each condition. Representative experiment shown.

C. Cell growth in HCT116 cells with CRISPR KO p53 treated with either an HA-tagged MDM2 expression construct (MDM2) or a control vector (EV) and cultured in full media before being switched at day 1 to grow in serine and glycine-depleted media (or continued in full media). iRFP level in each well at end point in serine and glycine-depleted media (top) and iRFP intensity per well for all conditions at end point (bottom) shown for each cell line. Data are presented as mean ±SEM obtained from triplicate wells per condition. Representative experiment shown.

D. Cell growth in HCT116 cells with wild-type p53 treated with either an HA-tagged MDM2 expression construct (MDM2) or a control vector (EV) and cultured in full media before being switched at day 1 to grow in serine and glycine-depleted media (or continued in full media). iRFP level in each well at end point in serine and glycine-depleted media (top) and iRFP intensity per well for all conditions at end point (bottom) shown for each cell line. Data are presented as mean ±SEM obtained from triplicate wells

Sup. Figure 3

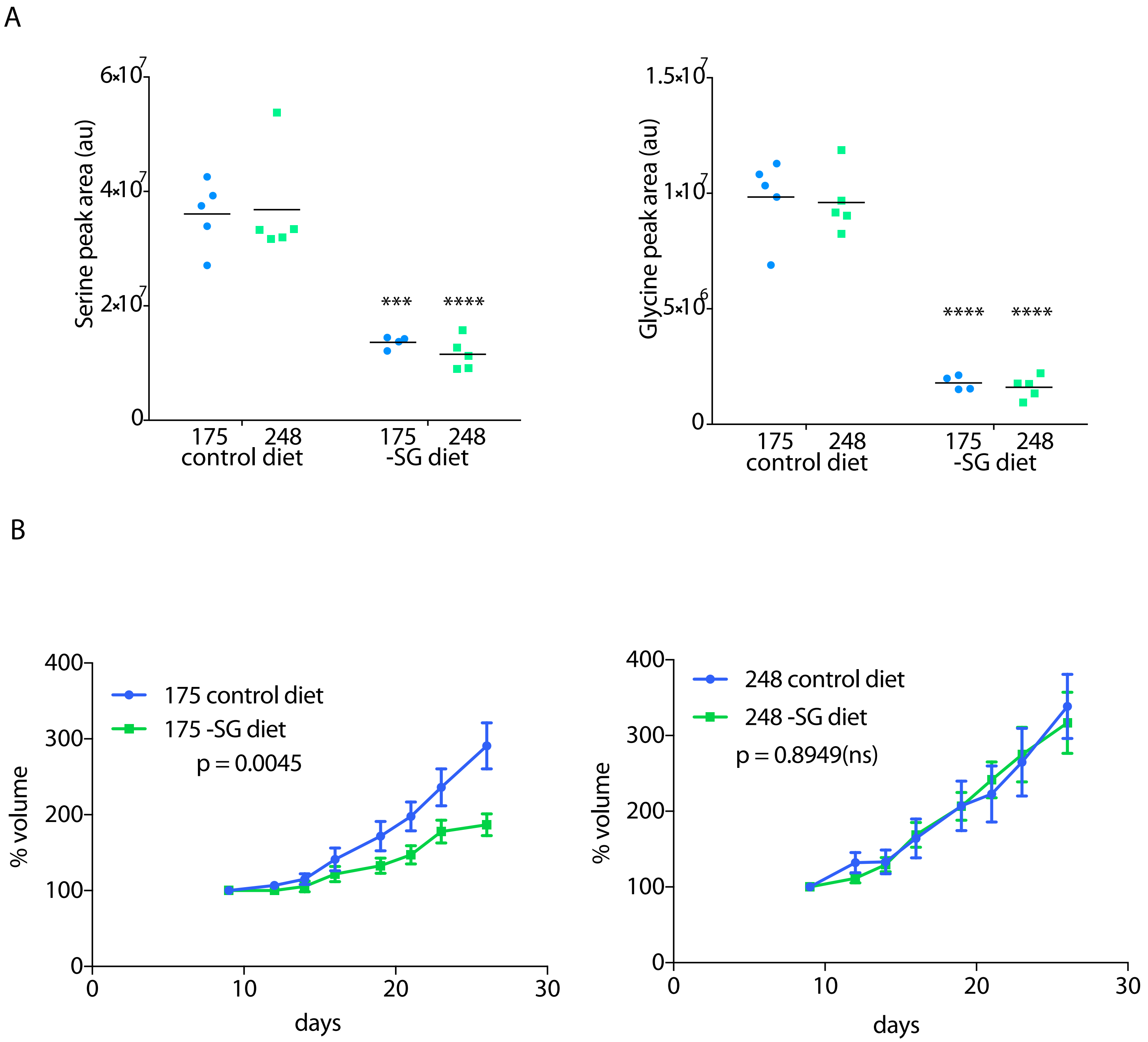

Figure S3: R248W mutant p53 confers enhanced tumour growth in serine and glycine-depleted conditions *in vivo*

A. Circulating levels of serine or glycine in the serum of experimental animals at endpoint of xenograft experiment as determined by LC-MS analysis. Each data point represents the mean peak area of duplicate samples from each mouse on the indicated diet (control or -SG) and injected with indicated tumour cell line (HCT116 with R175H or R248W mutant p53). Black bars represent mean per sample group. Data were analysed using a 2-way ANOVA with Holm-Sidak's multiple comparisons test and multiplicity-adjusted p-values.

B. Tumour growth in HCT116 cells expressing either R175H or R248W injected SC into immunocompromised mice fed either control or -SG diet. Tumour growth was monitored by calliper measurement at regular intervals as shown. Data are from the same mice and tumours as depicted in figure 4A (where iRFP measurements are shown). Data presented as tumour volume relative to day 9 baseline reading (mean ±SEM) for each condition from N= 9 175 control, N=8 175 -SG, N=8 248 control, and N=10 248 -SG tumours. Data analysed using a 2-way ANOVA with Tukey's multiple comparisons test and multiplicity-adjusted p-values. P-value reported in figure from final measurements.
